# iNKT Cells Suppress Pathogenic NK1.1^+^CD8^+^ T Cells in DSS-Induced Colitis

**DOI:** 10.3389/fimmu.2018.02168

**Published:** 2018-10-02

**Authors:** Sung Won Lee, Hyun Jung Park, Jae Hee Cheon, Lan Wu, Luc Van Kaer, Seokmann Hong

**Affiliations:** ^1^Department of Integrative Bioscience and Biotechnology, Institute of Anticancer Medicine Development, Sejong University, Seoul, South Korea; ^2^Department of Internal Medicine and Institute of Gastroenterology, Yonsei University College of Medicine, Seoul, South Korea; ^3^Department of Pathology, Microbiology and Immunology, Vanderbilt University School of Medicine, Nashville, TN, United States

**Keywords:** CD1d-dependent NKT cells, NK1.1^+^CD8^+^ T cells, Treg cells, IFNγ, DSS-induced colitis

## Abstract

T cells producing IFNγ play a pathogenic role in the development of inflammatory bowel disease (IBD). To investigate the functions of CD1d-dependent invariant natural killer T (iNKT) cells in experimental colitis induced in Yeti mice with dysregulated expression of IFNγ, we generated iNKT cell-deficient Yeti/CD1d KO mice and compared colitis among WT, CD1d KO, Yeti, and Yeti/CD1d KO mice following DSS treatment. We found that deficiency of iNKT cells exacerbated colitis and disease pathogenesis was mainly mediated by NK1.1^+^CD8^+^ T cells. Furthermore, the protective effects of iNKT cells correlated with up-regulation of regulatory T cells. Taken together, our results have demonstrated that CD1d-dependent iNKT cells and CD1d-independent NK1.1^+^CD8^+^ T cells reciprocally regulate the development of intestinal inflammatory responses mediated by IFNγ-dysregulation. These findings also identify NK1.1^+^CD8^+^ T cells as novel target cells for the development of therapeutics for human IBD.

## Introduction

Inflammatory bowel disease (IBD) is a chronic inflammatory disorder of the gastrointestinal tract that is caused by dysregulated immune responses to host intestinal bacterial flora (1). Human IBD can be divided into two major types, Crohn's disease and ulcerative colitis (UC), which share symptoms and treatment options but differ in their pathogenic features and expression profiles of inflammatory mediators. Crohn's disease is characterized by excessive T helper 1 (Th1) responses and involves pathogenic lesions in any part of the gastrointestinal tract, whereas UC is characterized by Th2-dominant conditions that result in lesions restricted to the colon and rectum (1). Moreover, it has been reported that increased Th17 immune responses are also associated with the progression of both Crohn's disease and UC (2). In mice, IBD can be acutely induced by oral administration of dextran sulfate sodium (DSS) and this colitis is characterized by elevated Th1 and Th17 cytokine responses.

The development of colitis involves a variety of immune cells, including dendritic cells (DCs), macrophages, monocytes, neutrophils, conventional αβ T cells, γδ T cells, natural killer (NK) cells, natural killer T (NKT) cells, and innate lymphoid cells (ILC) that have been suggested to play either protective or pathogenic roles (3–5). In particular, NKT cells, defined by expression of both NK cell markers (NK1.1 or DX5) and T cell markers (TCRβ or CD3) and restriction by the MHC class I-related protein CD1d, display several subsets with distinct cytokine profiles in gastrointestinal lymphoid tissues, including Peyer's patches (PP), lamina propria (LP), and mesenteric lymph nodes (MLN) of the small and large intestine (6). A role of CD1d-restricted type I NKT cells, also called invariant NKT (iNKT) cells because of the expression of an invariant TCR α chain (Vα14-Jα18 in mice and Vα24-Jα18 in humans), in regulating the development of IBD is well-established. For example, iNKT cells secreting IL13 play pathogenic roles in the oxazolone-induced UC mouse model (7). In contrast, adoptive transfer of IL9-producing iNKT cells (8) or administration of iNKT cell ligands (9, 10) exerted protective effects against DSS-induced colitis. In addition, roles for CD1d-restricted type II and DX5^+^ NKT cells in preventing the pathogenesis of IBD have been reported (11, 12). Additionally, CD1d-independent NK1.1^+^ T cells have been identified in the intestinal tissues of CD1d knockout (KO) mice (13, 14), but their potential contribution to the pathogenesis of colitis has not been explored.

Previously, Yeti mice were generated to monitor IFNγ-expressing immune cells *in vivo* by targeting an IRES/yellow fluorescent protein (YFP) reporter cassette downstream of the endogenous *ifng* gene (15). However, these Yeti mice have recently been reported to display autoinflammatory syndromes mediated by chronically elevated levels of IFNγ due to enhanced stability of IFNγ mRNA transcripts by using a polyA bovine growth hormone sequence (16). Thus, Yeti mice can be used to evaluate the role of IFNγ in chronic inflammatory conditions such as IBD.

Here, we have investigated the role of iNKT cells in colitis induced by DSS in Yeti mice with dysregulated IFNγ-mediated intestinal inflammation. We found that CD1d-deficiency exacerbated intestinal inflammation in these animals. Moreover, we found that disease in these animals was predominantly mediated by NK1.1^+^CD8^+^ T cells. Furthermore, we found that disease suppression mediated by iNKT cells was linked with the expansion of Foxp3^+^ regulatory T (Treg) cells.

## Materials and methods

### Mice

Wild-type (WT) C57BL/6 (B6) mice were purchased from Jung Ang Lab Animal Inc. (Seoul, Korea). IFNγ/YFP (Yeti) cytokine reporter mice were kindly provided by Dr. R. Locksley (University of California at San Francisco, CA, USA). CD1d KO mice were provided by Dr. A. Bendelac (University of Chicago, IL, USA). Jα18 KO mice were provided by Dr. M. Taniguchi (RIKEN, Yokohama, Japan). Yeti mice were further crossed with either CD1d KO or Jα18 KO mice to obtain Yeti/CD1d KO and Yeti/Jα18 KO mice, respectively. All mice in this study were on a B6 genetic background, were maintained at Sejong University, and were used for experiments at 6–12 weeks of age. They were maintained on a 12-h light/12-h dark cycle in a temperature-controlled barrier facility with free access to food and water. Mice were fed a γ-irradiated sterile diet and provided with autoclaved tap water. Age- and sex-matched mice were used for all experiments. The animal experiments were approved by the Institutional Animal Care and Use Committee at Sejong University (SJ-20160704).

### Induction of colonic inflammation

Mice were provided with 1.5% (w/v) DSS in the drinking water for 5 days. Subsequently, groups of mice were given normal control water for 5 days until sacrifice for experiments. To evaluate the clinical symptoms of DSS-induced colitis, the mice were monitored for a change in the percentage of body weight (0, none; 1, 1–10%; 2, 11–20%; 3, >20%), stool consistency (0, normal; 1, loose stool; 2, diarrhea), and bleeding (0, normal; 1, hemoccult positive; 2, gross bleeding) on a daily basis during colitis induction for 10 days. The body weight was expressed as a percentage of weight change for each individual mouse and was calculated relative to the starting body weight on day 0. These data were used to calculate a disease activity index (DAI).

### Cell culture and cell enrichment by magnetically activated cell sorting (MACS)

A single-cell suspension of splenocytes was prepared and resuspended in RPMI complete medium consisting of RPMI 1640 (Gibco BRL, USA) medium supplemented with 10% FBS, 10 mM HEPES, 2 mM L-glutamine, 100 units/mL penicillin-streptomycin, and 5 mM 2-mercaptoethanol. Naive CD4^+^ T cells from Jα18 KO B6 mice were enriched with the CD4^+^CD62L^+^ T cell isolation kit II (Miltenyi Biotech, Bergisch Gladbach, Germany), following the manufacturer's instructions. The naive CD4^+^ T cells were >94% pure among all MACS-purified populations. iNKT cells were enriched using NK1.1^+^ iNKT cell isolation kit (Miltenyi Biotech) following the manufacturer's instructions. The NKT cell population was >89% pure among all MACS-purified populations. CD8^+^ T cells that include NK1.1^+^CD8^+^ T cells but lack CD1d-dependent NKT cells were enriched from MLN cells isolated from Yeti/CD1d KO mice by negative selection of CD11c^+^ cells using anti-CD11c MACS and LD column, followed by positive selection with the CD8^+^ T cell MACS system. NK1.1^−^CD8^+^ T cells were enriched from MLN cells isolated from Yeti/CD1d KO mice by first removing NK1.1^+^ cells and CD11c^+^ cells using anti-CD11c MACS and anti-PE MACS after staining with PE-conjugated anti-NK1.1 (clone PK-136) mAb and LD column, followed by positive selection with the CD8^+^ T cell MACS system. Cell populations included >95% CD8^+^ cells among all MACS-purified populations. IL15-cultured NK1.1^+^CD8^+^ T cells from CD1d KO MLN were separated using Lympholyte-M (Cedar Lane Laboratories Ltd., Hornby, Ontario, Canada) by density gradient centrifugation and further positively selected for the NK1.1^+^ population using anti-PE MACS after staining with PE-conjugated anti-NK1.1 (clone PK-136) mAb. The NK1.1^+^CD8^+^ T cell population was >91% pure among all MACS-purified populations.

### *In vitro* CD4^+^ and CD8^+^ T cell differentiation

Recombinant murine IL15 and human TGFβ were purchased from R&D systems (Minneapolis, MN, USA) and recombinant murine IL2 was purchased from Peprotech (Hamburg, Germany). For *in vitro* stimulation, rIL15, rTGFβ, and rIL2 were used at a concentration of 10 ng/ml. Lipopolysaccharide (LPS) derived from *E. coli* (serotype 0111:B4) was purchased from Sigma-Aldrich (St. Louis, MO, USA). For CD4^+^ T cell differentiation, anti-IFNγ mAbs (5 μg/ml; clone XMG1.2, BD Biosciences) were added at the concentrations indicated in figure legends. Isolated naive CD4^+^CD62L^+^ T cells (1 × 10^5^ cells/well) were cultured with 96-well plate-bound anti-CD3ε (10 μg/ml) + anti-CD28 (1 μg/ml) mAbs in the presence of rTGFβ (10 ng/ml) and rIL2 (10 ng/ml) for 5 days. For CD8^+^ T cell differentiation, MLN NK1.1^+^CD8^+^ T cell-depleted CD8^+^ T cells (5 × 10^5^ cells/ml) isolated from either CD1d KO or Yeti/CD1d KO mice were cultured with rIL15 (10 ng/ml) in 24-well plates for 5 or 10 days.

### Flow cytometry

The following monoclonal antibodies (mAbs) were obtained from BD Biosciences (San Jose, USA): phycoerythrin (PE)-, or allophycocyanin (APC)-conjugated anti-NK1.1 (clone PK-136); PE-Cy7-, or APC-conjugated anti-CD4 (clone RM4-5); PE-Cy7-conjugated anti-TCRβ (clone H57-597); PE-Cy7-conjugated anti-CD8α (clone 53-6.7); PE-Cy7-, or APC-conjugated anti-CD3ε (clone 145-2C11); PE-Cy7-conjugated anti-CD11b (clone M1/70); APC-conjugated anti-CD25 (clone PC61); APC-conjugated anti-CD44 (clone IM7); APC-conjugated anti-CD314 (NKG2D) (clone CX5); PE-conjugated anti-IL12p40 (clone C15.6); PE-conjugated anti-TNFα (clone XP6-XT22); and PE-conjugated anti-IgG1 (κ isotype control).

The following mAbs from eBioscience were used: PE-conjugated anti-Ly49A (clone A1); PE-conjugated anti-FasL (clone MFL3); APC-conjugated anti-F4/80 (clone BM8); PE-conjugated anti-Perforin (clone eBioOMAK-D); PE-conjugated anti-IFNγ (clone XMG1.2); PE-conjugated anti-IL17A (clone eBio17B7); PE-conjugated anti-Eomes (clone Dan11mag); and PE-conjugated anti-Foxp3 (clone NRRF-30). The following mAb from R&D Systems was used: Biotin-conjugated anti-IL15. Flow cytometric data were acquired with a FACSCalibur system (Becton Dickinson, USA) and analyzed with FlowJo software (Tree Star, USA).

For surface antibody staining, cells were harvested and washed twice with cold 0.5% BSA-containing PBS (FACS buffer). For blocking non-specific binding to Fc receptors, the cells were incubated with anti-CD16/CD32 mAbs on ice for 10 min and subsequently stained with fluorescence-labeled mAbs. Flow cytometric data were acquired using a FACSCalibur flow cytometer (Becton Dickson, San Jose, CA, USA) and analyzed using FlowJo software (Tree Star Inc., Ashland, OR, USA).

### Intracellular cytokine staining

For intracellular staining, splenocytes were incubated with brefeldin A, an intracellular protein transport inhibitor (10 μg/ml), in RPMI medium for 2 h at 37°C. The cells were stained for cell surface markers, fixed with 1% paraformaldehyde, washed once with cold FACS buffer, and permeabilized with 0.5% saponin. The permeabilized cells were then stained for an additional 30 min at room temperature with the indicated mAbs (PE-conjugated anti-IL12p40, anti-IFNγ, anti-IL17A, and anti-perforin; PE-conjugated isotype control rat IgG mAbs). Fixation and permeabilization were performed using a Foxp3 staining kit (eBioscience) with the indicated mAbs (FITC-conjugated anti-Foxp3; PE-conjugated anti-Foxp3; FITC- or PE-conjugated isotype control rat IgG mAbs). More than 5,000 cells per sample were acquired using a FACSCalibur, and the data were analyzed using the FlowJo software package (Tree Star, Ashland, OR, USA).

### Isolation of colon MLN, IEL, and LP leukocytes

The MLN were aseptically removed, and single-cell suspensions of the MLN were obtained by homogenization and passing through a 70 μm nylon cell strainer. The large intestines were removed and flushed with 20 ml of cold CMF solution (Ca^2+^-Mg^2+^-free PBS containing 10 mM HEPES, 25 mM sodium bicarbonate, and 2% FBS). After exclusion of PP, fat, and mucus, the large intestine was cut longitudinally into 5 mm pieces and the pieces of tissue were washed twice with CMF solution. The tissues were transferred into 15 ml pre-warmed EDTA/DTT/FBS/CMF solution (CMF containing 1 mM EDTA, 1 mM DTT, 10% FBS, and 100 units/mL penicillin-streptomycin) and stirred in 37°C shaking/orbital incubator for 30 min to obtain intraepithelial lymphocytes (IEL). The cell-containing suspension was passed through a 70 μm nylon cell strainer (BD Falcon), and put on ice. IELs were purified from the interface of a 40/70% Percoll (GE Healthcare) gradient after centrifugation for 20 min at 2,400 rpm at RT. To isolate the lamina propria lymphocytes (LPLs), the remaining intestinal tissues were cut into small pieces using a scalpel and transferred to conical tubes. Tissues were resuspended in 20 ml of complete RPMI containing 2.5 mg/ml collagenase type IV (Sigma, St. Louis, MO, USA) and 1 mg/ml DNase I (Promega, Madison, USA) and shaken at 200 rpm for 40 min at 37°C. At the end of the incubation, the digested tissues were dissociated into single-cell suspensions using gentle MACS Dissociator (Miltenyi, Germany) in combination with C Tubes. The cell-containing suspension was passed through a 70 μm nylon cell strainer (BD Falcon), and put on ice. LPLs were purified from the interface of a 40/70% Percoll (GE Healthcare) gradient after centrifugation for 20 min at 2,400 rpm at RT.

### Histology

Distal colonic sections were fixed in 4% paraformaldehyde, embedded in paraffin, and cut into 6 μm sections using a microtome (RM 2235, Leica, Germany). The sections were then stained with H&E for the analysis of histological changes. The histological score of each individual mouse was measured as follows: epithelial damage (E), 0 = none; 1 = minimal loss of goblet cells; 2 = extensive loss of goblet cells; 3 = minimal loss of crypts and extensive loss of goblet cells; 4 = extensive loss of crypts; and infiltration (I), 0 = no infiltrate; 1 = infiltrate around the crypt basis; 2 = infiltrate reaching the muscularis mucosa; 3 = extensive infiltration reaching the muscularis mucosa and thickening of the mucosa with abundant edema; 4 = infiltration of the submucosa. The total histological score was calculated as E+I.

### Cytotoxicity assay

The flow cytometric CFSE/7-AAD cytotoxicity assay was performed as previously described (17) with minor modifications. NK1.1^−^CD8^+^ T cells and NK1.1^+^CD8^+^ T cells were isolated as described above and suspended in complete RPMI medium. B16 melanoma cells (3 × 10^6^) were labeled with 500 nM CFSE in Hanks' Balanced Salt Solution for 10 min at 37°C in a volume of 2 ml. The cells were washed twice in RPMI medium and used immediately. The CFSE-labeled target cells (20,000 cells) were incubated with either NK1.1^−^CD8^+^ T cells or NK1.1^+^CD8^+^ T cells at different effector (E): target (T) ratios (0:1, 3:1, 9:1, and 27:1). After 10 h of incubation, cells were stained with 0.25 μg/ml of 7-AAD and were incubated for 10 min at 37°C in a CO_2_ incubator. Cells were washed twice with 1 × PBS containing 1% FBS (FACS buffer) and resuspended in FACS buffer. Cytotoxicity was assessed by flow cytometry.

### Statistical analysis

Statistical significance was determined using Excel (Microsoft, USA). Student's *t*-test was performed for the comparison of two groups. ^*^P < 0.05, ^**^P < 0.01, and ^***^P < 0.001 were considered to be significant in the Student's *t*-test. Two-way ANOVA analysis was carried out using the VassarStats (http://vassarstats.net/anova2u.html). ^#^*P* < 0.05, ^##^*P* < 0.01, and ^###^*P* < 0.001 were considered to be significant in the two-way ANOVA.

## Results

### Yeti mice have increased numbers of CD1d-independent NK1.1^+^CD8^+^ T cells

**Figure 1:**
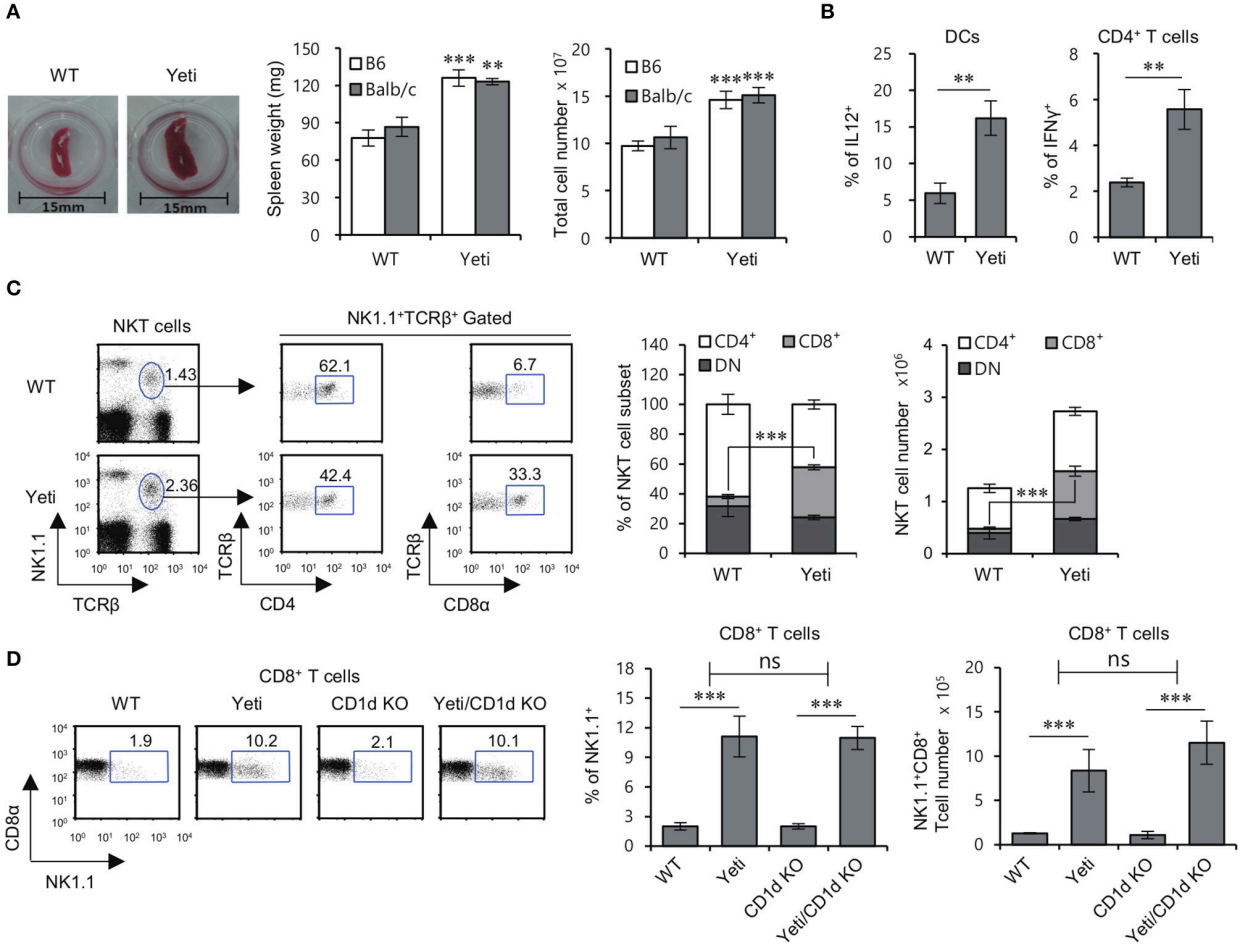
Altered NKT cell subsets in Yeti mice. **(A)** Left, a representative picture of the spleens from 8-week-old WT B6 and heterozygous Yeti B6 mice. Middle and Right, Spleen weight and splenocyte number in Yeti B6 and Yeti Balb/c mice, as compared with WT B6 and Balb/c mice. **(B)** Intracellular IL12 production by isolated DCs (CD11c^+^) from WT B6 and Yeti B6 mice was assessed by flow cytometry. Intracellular IFNγ production was assessed in splenic CD4^+^ T cells from WT B6 or Yeti B6 mice by flow cytometry. **(C)** The percentage of NK1.1^+^TCRβ^+^ cells among splenocytes and the percentage of either CD4^+^ or CD8α^+^ populations among NK1.1^+^ T cells from 8-week-old WT and Yeti mice are plotted. The proportion and absolute cell numbers of CD4^+^, CD8^+^, and DN NK1.1^+^ T cells were assessed in WT and Yeti mice at the age of 8 weeks. **(D)** The percentage of NK1.1^+^ populations among CD8α^+^ T cells from 8-week-old WT, Yeti, CD1d KO, and Yeti/CD1d KO mice are plotted. The mean values ± SD (*n* = 4 per group in the experiment; Student's *t*-test; ^**^P < 0.01, ^***^P < 0.001) are shown. Two-way ANOVA (Yeti × iNKT) showed an interaction between these two factors (ns, not significant).

Since it has been reported that IFNγ KO mice are protected from DSS-induced colitis (18), we examined whether dysregulated IFNγ expression in heterozygous Yeti mice, in which abnormal IFNγ secretion occurs due to modification of the 3′-untranslated region (UTR) of the IFNγ gene (16), influences colitis severity. We confirmed that heterozygous Yeti mice display splenomegaly and elevated levels of IL12-producing DCs and Th1 cells compared with WT mice (Figures 1A,B).

While studying NKT cells in Yeti mice, we noticed that the proportion and absolute number of NK1.1^+^CD8^+^ T cells among total splenocytes were increased five- to eight-fold in Yeti mice compared with WT mice. Moreover, a similar increase in NK1.1^+^CD8^+^ T cells in Yeti mice was observed in NKT cell-deficient Yeti/CD1d KO mice, consistent with the notion that these cells are not CD1d-restricted (Figures 1C,D). Collectively, these results suggest that the excessive inflammation in Yeti mice might be associated with an increase in the numbers of NK1.1^+^CD8^+^ T cells.

### Yeti mice accelerate intestinal inflammation in the absence of iNKT cells

**Figure 2:**
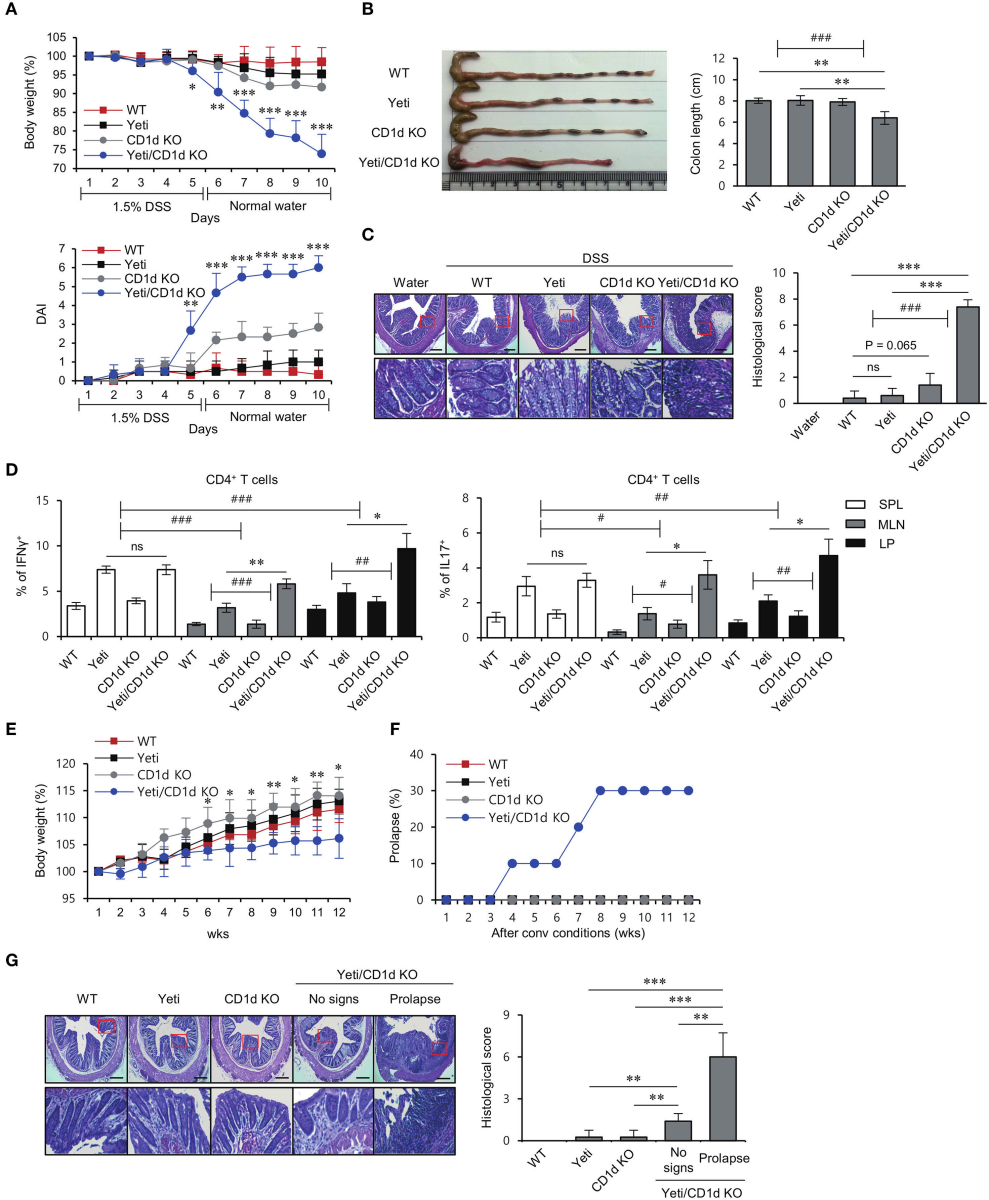
Lack of iNKT cells accelerates intestinal inflammation in Yeti mice. Daily body weight changes, DAI score **(A)** and colon length **(B)** of WT, Yeti, CD1d KO, and Yeti/CD1d KO mice were evaluated after 1.5% DSS treatment. Data are representative of three independent experiments with similar results. **(C)** On day 10, distal colons from each group were sectioned and stained with H&E. **(D)** Intracellular IFNγ and IL17 production were assessed in splenic, MLN, and LP CD4^+^ T cells from these mice by flow cytometry on day 10. The mean values ± SD (n = 5 per group in the experiment; Student's *t*-test; ^*^P < 0.05, ^**^P < 0.01, ^***^P < 0.001) are shown. Two-way ANOVA (Yeti × iNKT and genotype × tissue) showed an interaction between these two factors (^#^*P* < 0.05, ^*##*^*P* < 0.01, ^*###*^*P* < 0.001). Daily body weight changes **(E)** and prolapse rate **(F)** of WT, Yeti, CD1d KO, and Yeti/CD1d KO mice housed for 12 weeks under conventional conditions. (*n* = 7 for WT, Yeti, and CD1d KO mice; *n* = 10 for Yeti/CD1d KO mice; Student's *t*-test; ^*^P < 0.05, ^**^P < 0.01). **(G)** Left, distal colons from these mice were sectioned and stained with H&E at week 12. Right, histologic damages were scored from H&E-stained sections at week 12. (*n* = 5 for WT, Yeti, and CD1d KO mice; *n* = 5 for Yeti/CD1d KO mice (no signs); *n* = 3 for Yeti/CD1d KO (prolapse); Student's *t*-test; ^**^P < 0.01, ^***^P < 0.001).

Since iNKT cells can provide protective effects against DSS-induced colitis (8, 9), we decided to examine whether these cells play a role in regulating intestinal inflammation in Yeti mice. To address this possibility, we generated iNKT cell-deficient Yeti/CD1d KO mice by crossing Yeti mice with CD1d KO mice. We compared the colitic symptoms among WT, CD1d KO, Yeti, and Yeti/CD1d KO mice following 1.5% DSS treatment. Yeti/CD1d KO mice showed more severe weight loss, diarrhea, and bleeding in feces, resulting in a steep increase in the DAI score, compared with the different groups of control mice (Figure 2A). Furthermore, the colon length in DSS-treated Yeti/CD1d KO mice was remarkably shortened (Figure 2B) and displayed increased signs of colonic inflammation, with loss of epithelial crypts, edema, and infiltration of inflammatory cells (Figure 2C). In addition, we found that Th1 and Th17 differentiation of CD4^+^ T cells in the spleen, MLN, and LP from Yeti and Yeti/CD1d KO mice was significantly increased as compared with WT and CD1d KO mice after DSS administration. The frequencies of Th1 and Th17 cells in Yeti/CD1d KO mice were two- to three-fold greater in the MLN and LP, but not in the spleen, than those from Yeti mice, indicating that the protective role of iNKT cells in colitis was largely restricted to the MLN and LP (Figure 2D). On the other hand, colitis in CD1d KO mice was only slightly increased compared with WT mice, suggesting that lack of iNKT cells by itself is not sufficient to induce exacerbated colitis.

Since CD1d KO mice lack iNKT (Type I) cells as well as type II NKT cells, we generated Yeti/Jα18 KO mice by crossing Yeti mice with Jα18 KO mice that selectively lack iNKT cells. Upon administration of DSS, Yeti/Jα18 KO mice showed significantly higher disease activity than the control mice (Supplementary Figures 1A,B). Taken together, these results indicate that CD1d-dependent iNKT cells play a major role in controlling DSS-induced colitis in Yeti mice.

IL10 KO and IL2 KO mice spontaneously develop colitis when raised under conventional conditions by dysregulated immune responses against commensal bacteria (19, 20). To investigate whether WT, CD1d KO, Yeti, and Yeti/CD1d KO mice develop intestinal inflammation spontaneously when raised under conventional conditions, we measured body weights once a week for 12 weeks. We observed that Yeti/CD1d KO mice gained body weight more slowly compared with the other groups of mice (Figure 2E). Furthermore, unexpectedly, the incidence of rectal prolapse was significantly increased in Yeti/CD1d KO mice (Figure 2F). This was confirmed by histological examination of H&E-stained colon sections (Figure 2G). Taken together, these results suggest that colitic pathogenesis in Yeti/CD1d KO mice is mainly attributed to both deficiency of iNKT cells and increase of NK1.1^+^CD8^+^ T cells.

### NK1.1^+^CD8^+^ T cells with effector/memory phenotypes strongly correlate with severe intestinal inflammation in yeti mice

**Figure 3:**
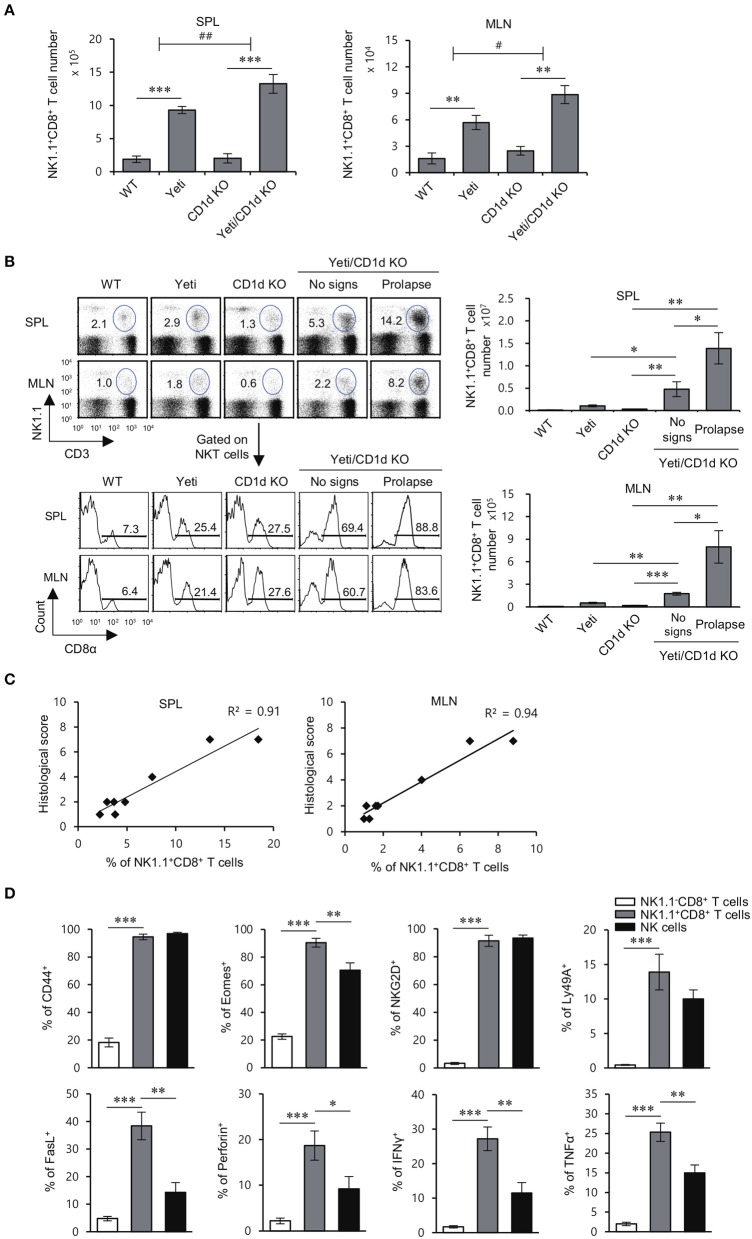
The development of colitis in Yeti/CD1d KO mice is associated with increased numbers of NK1.1^+^CD8^+^ T cells. The spleen and MLN were obtained from 1.5% DSS-treated WT, Yeti, CD1d KO, and Yeti/CD1d KO mice at day 10. **(A)** The absolute numbers of NK1.1^+^CD8^+^ T cells in the indicated tissues from these mice were assessed by flow cytometry at day 10. The mean values ± SD (*n* = 5 per group in the experiment; Student's *t*-test; ^**^P < 0.01, ^***^P < 0.001) are shown. Two-way ANOVA (Yeti × iNKT) showed an interaction between these two factors (^#^*P* < 0.05, ^*##*^*P* < 0.01). **(B,C)** The spleen and MLN were isolated from WT, Yeti, CD1d KO, and Yeti/CD1d KO mice at 12 weeks after initiation of conventional housing conditions. **(B)** Upper left, the percentage of NK1.1^+^CD3^+^ cells among splenocytes and MLN cells of these mice was determined at week 12. Lower left, the frequency of the CD8α^+^ population among NK1.1^+^CD3^+^ cells from the spleen and MLN of these mice was determined at week 12. Right, the absolute numbers of NK1.1^+^CD8^+^ T cells in the spleen and MLN were assessed by flow cytometry at week 12. (*n* = 5 for WT, Yeti, and CD1d KO mice; *n* = 5 for Yeti/CD1d KO mice (no signs); *n* = 3 for Yeti/CD1d KO (prolapse); Student's *t*-test; ^*^P < 0.05, ^**^P < 0.01, ^***^P < 0.001). **(C)** Scatter graphs and linear regression analysis of the relationship between the frequency of NK1.1^+^CD8^+^ T cells among total splenocytes or MLN cells and histological score of colon tissue sections in Yeti/CD1d KO mice. The Pearson's correlation coefficient (*R*^2^) for each plot is indicated. (*n* = 5 for Yeti/CD1d KO mice (no signs); *n* = 3 for Yeti/CD1d KO (prolapse)). **(D)** The expression of CD44, Eomes, NKG2D, Ly49a, FasL, perforin, IFNγ, and TNFα among NK1.1^−^CD8^+^ T cells, NK1.1^+^CD8^+^ T cells, and NK cells of the MLN from 1.5% DSS-treated Yeti/CD1d KO mice was evaluated by flow cytometry on day 10. The mean values ± SD (*n* = 4 in **A–C**; *n* = 5 in **D**; per group in the experiment; Student's *t*-test; ^*^P < 0.05, ^**^P < 0.01, ^***^P < 0.001) are shown.

Since our results showed that the numbers of NK1.1^+^CD8^+^ T cells were increased in Yeti mice at steady state, we investigated whether NK1.1^+^CD8^+^ T cells are closely related with the intestinal inflammatory processes in Yeti mice. In DSS-induced colitis, the number of NK1.1^+^CD8^+^ T cells in the spleen and MLN from Yeti mice was expanded approximately 6-fold compared with WT mice, and the number of NK1.1^+^CD8^+^ T cells from Yeti/CD1d KO mice was increased eight-fold compared with CD1d KO mice (Figure 3A). Moreover, when colitis was spontaneously induced, the number of NK1.1^+^CD8^+^ T cells in the spleen and MLN from Yeti/CD1d KO mice with a prolapse were three- to four-fold higher compared to those from Yeti/CD1d KO mice without prolapse (Figure 3B), which strongly indicates that NK1.1^+^CD8^+^ T cells are pathogenic effector cells. Moreover, there was a strong positive correlation between NK1.1^+^CD8^+^ T cell number and the histological score of colon tissue samples in both the spleen (*R*^2^ = 0.91) and MLN (*R*^2^ = 0.94) (Figure 3C). Taken together, these findings suggest that NK1.1^+^CD8^+^ T cells are pathogenic cells contributing to severe and spontaneous colitis in Yeti/CD1d KO mice.

In previous reports, NK1.1-expressing CD8^+^ T cells were shown to display significant cytotoxicity against tumor cells and to possess a memory phenotype with a pro-inflammatory cytokine production profile (21, 22). Thus, we examined the expression of memory markers (CD44 and Eomes), NK cell receptors (NKG2D and Ly49a), cytolytic molecules (FasL and Perforin), and pro-inflammatory cytokines (IFNγ and TNFα) in MLN-derived NK1.1^+^CD8^+^ T cells from Yeti/CD1d KO mice during DSS-induced colitis. We found that NK1.1^+^CD8^+^ T cells from DSS-treated mice, unlike unstimulated NK1.1^−^CD8^+^ T cells, exhibit an effector CD8^+^ T cell phenotype with NK cell-like functions, suggesting that NK1.1^+^CD8^+^ T cells are pathogenic cells causing severe intestinal inflammation in Yeti mice (Figure 3D).

### The NK1.1^+^CD8^+^ T cell population is primarily responsible for induction of DSS colitis in yeti mice

**Figure 4:**
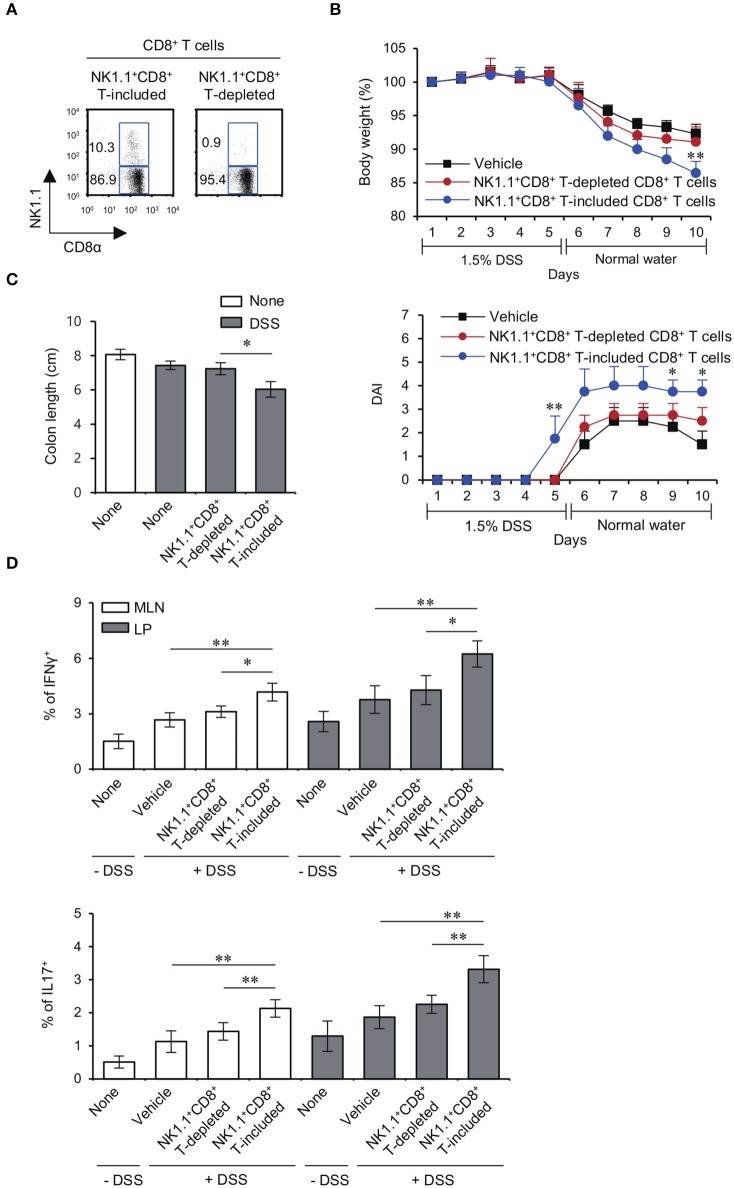
The NK1.1^+^ population among CD8^+^ T cells is mainly responsible for colitis in Yeti mice. **(A)** The percentage of NK1.1^+^CD8^+^ T cells among total CD8^+^ T cells and NK1.1^+^ cell-depleted CD8^+^ T cells from the MLN of Yeti/CD1d KO mice was determined. **(B–D)** Either total CD8^+^ T cells (2 × 10^6^) or NK1.1^−^CD8^+^ T cells (2 × 10^6^) from Yeti/CD1d KO mice were i.v. transferred to CD1d KO mice. Daily body weight changes, DAI score **(B)**, and colon length **(C)** of each group were evaluated after 1.5% DSS treatment. **(D)** Intracellular IFNγ and IL17 production and the frequencies of Foxp3^+^CD25^+^ cells were assessed in the MLN and LP CD4^+^ T cells from these mice by flow cytometry on day 10. The mean values ± SD (*n* = 5 per group in the experiment; Student's *t*-test; ^*^P < 0.05, ^**^P < 0.01) are shown.

It has been reported that colitogenic CD8^+^ T cells have an effector phenotype with increased expression of IFNγ and Granzyme B (23). Since the increased NK1.1^+^ population among Yeti CD8^+^ T cells produced high levels of pro-inflammatory cytokines and cytolytic molecules, we decided to examine whether the pathogenic capacity of Yeti CD8^+^ T cells was mediated by the NK1.1^+^ subpopulation. To test this possibility, we adoptively transferred MLN total CD8^+^ T cells or MLN NK1.1^−^CD8^+^ T cells from Yeti/CD1d KO into CD1d KO mice and treated these animals with 1.5% DSS (Figure 4A). Mice that received total CD8^+^ T cells showed progressive symptoms of colitis compared with uninjected mice, whereas disease in mice that received NK1.1^−^CD8^+^ T cells was very similar to uninjected mice (Figures 4B,C). Moreover, mice that received NK1.1^−^CD8^+^ T cells displayed a significant decrease in the frequencies of Th1 and Th17 cells in the MLN and LP compared to mice that received total CD8^+^ T cells including NK1.1^+^CD8^+^ T cells (Figure 4D). Overall, these results provide strong evidence that NK1.1^+^CD8^+^ T cells play a pivotal role in mediating the pathogenesis of DSS-induced colitis in Yeti mice.

### Lack of CD1d-restricted iNKT cells increases susceptibility to colitis induced by NK1.1^+^CD8^+^ T cells

**Figure 5:**
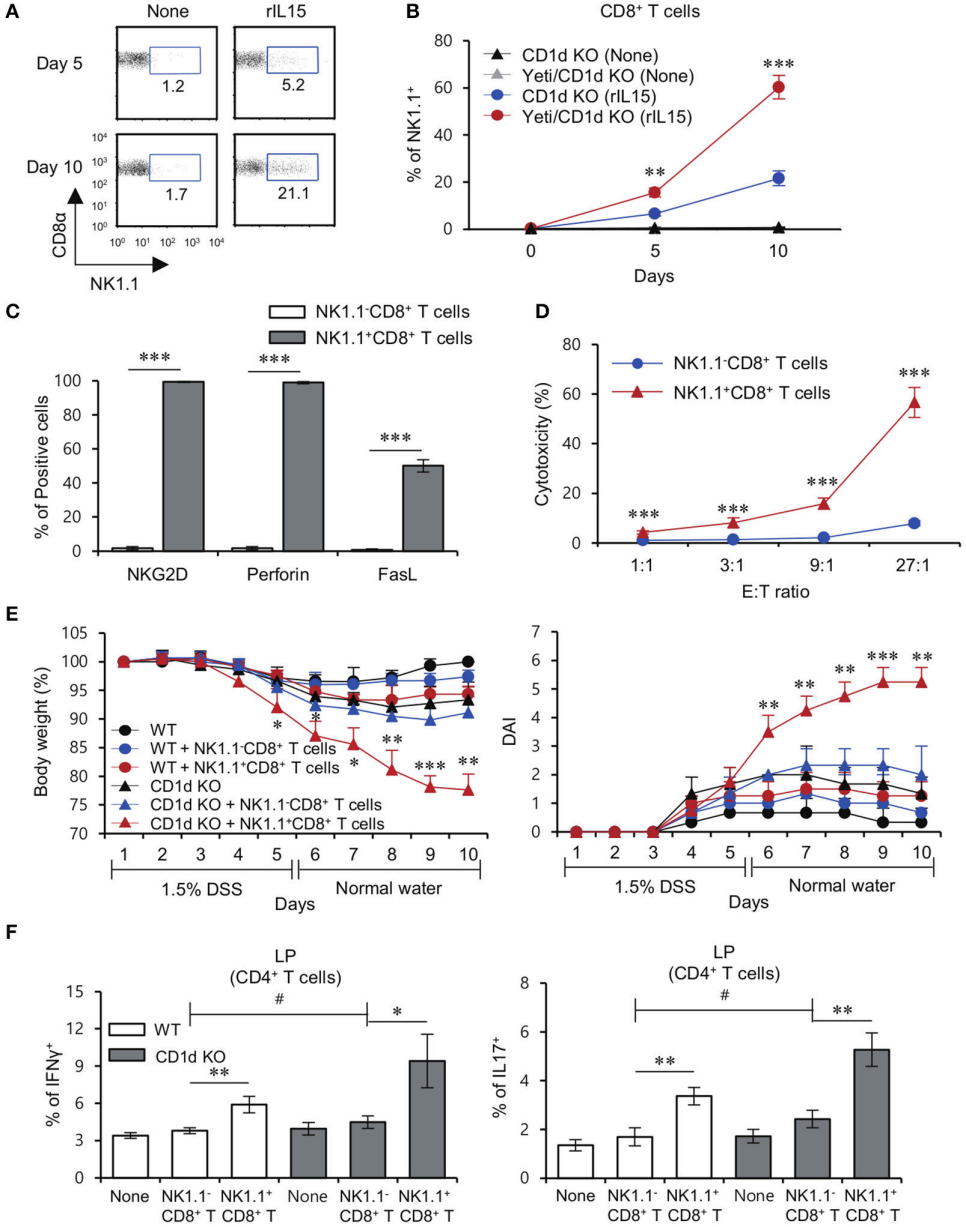
CD1d-restricted iNKT cells exhibit inhibitory effects on the pathogenesis of NK1.1^+^CD8^+^ T cell-mediated colitis. **(A)** Purified NK1.1^+^ cell-depleted CD8^+^ T cells from the MLN of CD1d KO mice were cultured with rIL15 for 5 or 10 days. The percentage of the NK1.1^+^ population among all CD8^+^ T cells was evaluated by flow cytometry at the indicated time points. **(B)** Purified NK1.1^−^CD8^+^ T cells from the MLN of CD1d KO and Yeti/CD1d KO mice were cultured with rIL15 for 5 or 10 days. The percentage of the NK1.1^+^ population among total CD8^+^ T cells was evaluated by flow cytometry at the indicated time points. **(C)** The expression of NKG2D, perforin, and FasL among unstimulated NK1.1^−^CD8^+^ T cells or IL15-stimulated NK1.1^+^CD8^+^ T cells were assessed by flow cytometry on day 10 after cytokine stimulation. **(D)** Cytotoxicity of either unstimulated NK1.1^−^CD8^+^ T cells or IL15-stimulated NK1.1^+^CD8^+^ T cells from the CD1d KO MLN was evaluated using 7-AAD/CFSE assay. **(E,F)** Either NK1.1^−^CD8^+^ T cells (1 × 10^6^) or IL15-stimulated NK1.1^+^CD8^+^ T cells (1 × 10^6^) from the CD1d KO MLN were i.v. transferred to CD1d KO mice. **(E)** Daily body weight changes and DAI score of each group were evaluated 10 days after 1.5% DSS treatment. **(F)** Intracellular IFNγ and IL17 production in LP CD4^+^ T cells from these mice were determined by flow cytometry on day 10. The mean values ± SD (*n* = 3 in **D**; *n* = 4 in **A–C**; *n* = 5 in **E** and **F**; per group in the experiment; Student's *t*-test; ^*^P < 0.05, ^**^P < 0.01, ^***^P < 0.001) are shown. Two-way ANOVA (genotype × treatment) showed an interaction between these two factors (^#^*P* < 0.05).

It has been reported that the common gamma chain (γc) cytokine IL15 directly induces NK1.1 expression on sorted CD8^+^ T cells (24). Consistent with this previous study, we found that NK1.1^−^CD8^+^ T cells cultured with IL15 significantly expressed higher levels of NK1.1 compared to cells cultured without cytokines (Figure 5A). Furthermore, we found that IFNγ-YFP reporter knockin increases NK1.1 expression on NK1.1^−^CD8^+^ T cells in response to IL15 stimulation (Figure 5B). We also found that IL15-induced NK1.1^+^CD8^+^ T cells expressed high levels of cytolytic molecules such as NKG2D, perforin, and FasL, indicating that IL15-induced NK1.1^+^CD8^+^ T cells display similar phenotypic profiles as Yeti NK1.1^+^CD8^+^ T cells (Figure 5C). Furthermore, we demonstrated that IL15-induced NK1.1^+^CD8^+^ T cells, unlike unstimulated NK1.1^−^CD8^+^ T cells, are effector cells with cytotoxic function against B16 melanoma cells (Figure 5D).

Previously, iNKT cells and NK1.1^+^CD8^+^ T cells have been implicated in the development of colitis (8, 14), but their interactions remain largely unknown. Importantly, adoptive transfer of IL15-induced NK1.1^+^CD8^+^ T cells resulted in a significant increase in DSS-induced weight loss and DAI score in the absence of iNKT cells, whereas DSS-induced colitis exacerbated by injection of IL15-induced NK1.1^+^CD8^+^ T cells was dramatically halted by the presence of iNKT cells (Figure 5E). Upon DSS-induced colitis, when compared with CD1d KO mice, WT mice that received NK1.1^+^CD8^+^ T cells showed significantly decreased frequency of Th1 and Th17 cells in the LP (Figure 5F). Collectively, these data demonstrate that NK1.1^+^CD8^+^ T cells are pathogenic in DSS colitis in the absence of iNKT cells.

### NK1.1^+^CD8^+^ T cells mediate inhibitory effects on treg cell differentiation in intestinal inflammation

**Figure 6:**
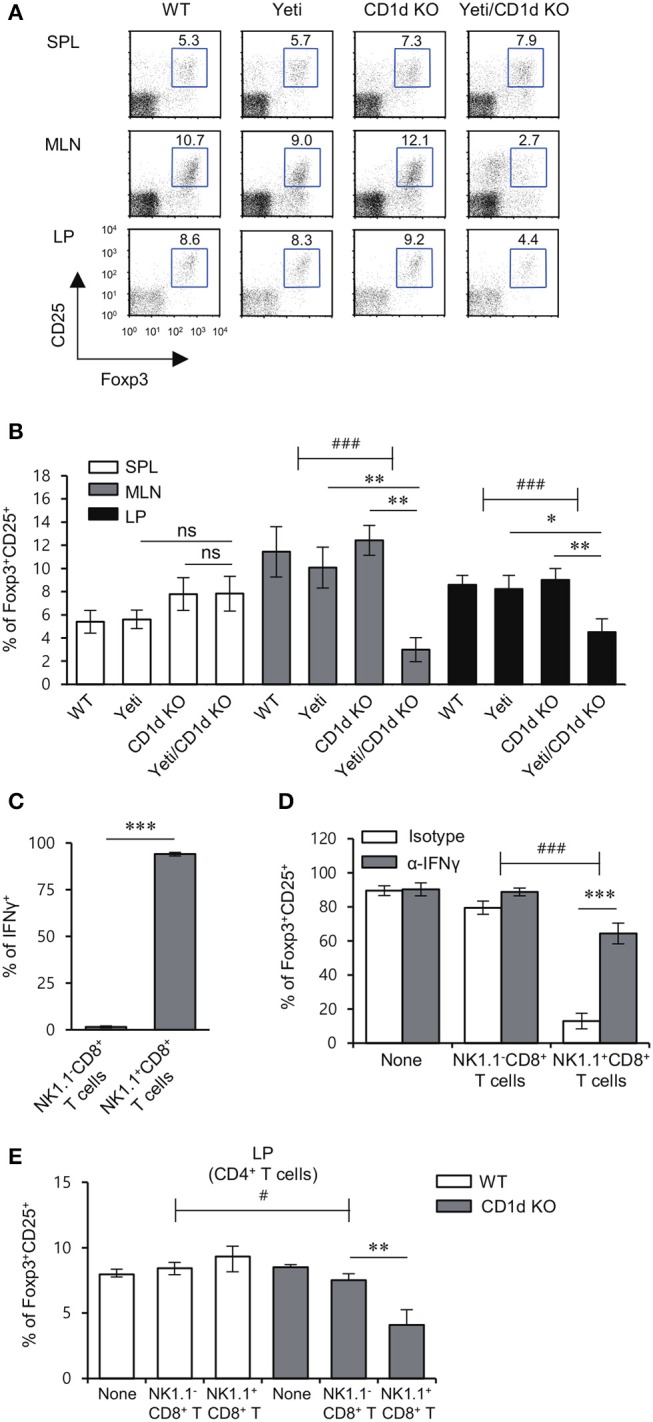
iNKT cells prevent the reduction in the Treg population induced by NK1.1^+^CD8^+^ T cells. **(A,B)** The spleen, MLN, and LP were obtained from WT, Yeti, CD1d KO, and Yeti/CD1d KO mice at 10 days after 1.5% DSS treatment. The percentage of Foxp3^+^CD25^+^ cells among CD4^+^ T cells from the spleen, MLN, and LP of each group was evaluated by flow cytometry on day 10. Data are representative of three independent experiments with similar results. The mean values ± SD (*n* = 5 per group in the experiment; Student's *t*-test; ^*^P < 0.05, ^**^P < 0.01) are shown. Two-way ANOVA (Yeti × iNKT) showed an interaction between these two factors (^*###*^*P* < 0.001). **(C)** The expression of IFNγ among unstimulated NK1.1^−^CD8^+^ T cells or IL15-stimulated NK1.1^+^CD8^+^ T cells were assessed by flow cytometry on day 10 after cytokine stimulation. **(D)** Naive CD4^+^CD62L^+^ T cells were co-cultured in Treg-polarizing conditions for 5 days with either unstimulated NK1.1^−^CD8^+^ T cells (1 × 10^4^ cells) or IL15-stimulated NK1.1^+^CD8^+^ T cells (1 × 10^4^ cells) from the CD1d KO MLN. Neutralizing mAb specific to IFNγ was added during the culture. The frequency of Foxp3^+^CD25^+^ cells among CD4^+^ T cells was evaluated by flow cytometry on day 5. Two-way ANOVA (treatment × cell type) showed an interaction between these two factors (^*###*^*P* < 0.001). **(E)** Either NK1.1^−^CD8^+^ T cells (1 × 10^6^) or IL15-stimulated NK1.1^+^CD8^+^ T cells (1 × 10^6^) from the CD1d KO MLN were i.v. transferred to CD1d KO mice. The frequencies of Foxp3^+^CD25^+^ cells in LP CD4^+^ T cells from these mice were determined by flow cytometry on day 10. The mean values ± SD (*n* = 4 in **C** and **D**; *n* = 5 in **E**; per group in the experiment; Student's *t*-test; ^*^P < 0.05, ^**^P < 0.01, ^***^P < 0.001) are shown. Two-way ANOVA (genotype × treatment) showed an interaction between these two factors (^#^*P* < 0.05).

The loss of colonic Treg cells induces excessive infiltration of inflammatory immune cells that results in colitis (25). A previous study demonstrated that IFNγ inhibits the generation of inducible Treg (iTreg) (26). In addition, activated NKT cells modulate Treg function in an IL2-dependent manner (27). Thus, we examined whether the lack of iNKT cells could affect the distribution of Treg cells in DSS-treated Yeti mice. Intriguingly, DSS-treated Yeti/CD1d KO mice contained fewer Treg cells in the MLN and LP, but not in the spleen when compared with WT, Yeti, and CD1d KO mice (Figures 6A,B). Taken together, these data suggest that the combined loss of iNKT cells and dysregulated IFNγ production can induce a decrease in protective Foxp3^+^CD25^+^CD4^+^ T cells during DSS-induced colitis. As expected, IL15-induced NK1.1^+^CD8^+^ T cells, unlike unstimulated NK1.1^−^CD8^+^ T cells, produced high levels of IFNγ (Figure 6C). Moreover, consistent with a previous study (28), our results showed that NK1.1^+^CD8^+^ T cell-derived IFNγ acts as a potent inhibitor of Treg cell differentiation (Figure 6D). Upon DSS-induced colitis, when compared with CD1d KO mice, WT mice that received NK1.1^+^CD8^+^ T cells displayed significantly increased frequency of Treg cells in the LP (Figure 6E). Collectively, these data strongly suggest that NK1.1^+^CD8^+^ T cells become pathogenic by suppressing Treg differentiation whereas iNKT cells can control the effects of NK1.1^+^CD8^+^ T cells on Treg cells during the development of colitis.

## Discussion

We have demonstrated here that the severity of low dose DSS (1.5%)-induced colitis is exacerbated in heterozygous Yeti mice on an NKT cell-deficient background, indicating that iNKT cells are essential for maintaining intestinal homeostasis in the context of IFNγ-mediated inflammation. We further showed that iNKT cells and Treg cells contribute to colitis protection, whereas expansion of CD1d-independent NK1.1^+^CD8^+^ T cells contributes to colitis pathogenesis.

Previously, Lee et al. suggested that an IL4-dominant environment in the MLN induced by oral administration of α-GalCer suppresses Th1-driven Crohn's disease but promotes Th2-driven ulcerative colitis (29). Based on these previous findings and our results reported here, the MLN might be a critical site for iNKT2 cell-mediated protection against DSS-induced colitis in Yeti mice. In addition to protein antigens that can activate conventional T cells, commensal bacteria contain glycolipid Ags, which are capable of activating iNKT cells through TCR ligation by Ag/CD1d complexes, but not TLR triggering (30). *Sphingomonas/Sphingobium* species, which contain glycolipid Ags, activate intestinal iNKT cells to produce cytokines in a CD1d-dependent manner (30), whereas intestinal microbe *Bacteroides fragilis*-derived glycolipid Ags inhibit iNKT cell activation through competitive binding with CD1d, resulting in protection against oxazolone-induced colitis (31). Thus, it is tempting to speculate that reduced survival of Treg cells in a Th1 cytokine-dominant environment triggered in Yeti mice might be inhibited by iNKT cells in response to glycolipid Ags derived from intestinal commensal bacteria.

Next, we investigated the cell types that contribute to disease pathogenesis in DSS-induced colitis under IFNγ dysregulated conditions. Since prior reports that CD1d-independent NK1.1^+^CD8^+^ T cells producing high levels of IFNγ and TNFα are involved in diverse immune responses such as tumor immune surveillance, allogeneic hematopoietic cell transplantation, and viral infection (21, 22, 32, 33), we considered the possibility that such cells might be involved. We found an expansion of NK1.1^+^CD8^+^ T cells during the development of DSS and spontaneous colitis in Yeti mice, and adoptive transfer experiments provided direct evidence for a pathogenic role of these cells in colitis.

Based on the previous report that IFNγ and IL12 do not directly up-regulate NK1.1 expression on the surface of CD8^+^ T cells (24), an increase in NK1.1-expressing CD8^+^ T cells might be attributed to enhanced IL15 secretion by mononuclear phagocytes (34–36), which is consistent with our observations that IL15 production in both DCs and macrophages from Yeti mice was significantly increased in both the spleen and MLN compared to WT mice, indicating that DCs and macrophages might be critical sources of IL15 to increase NK1.1^+^CD8^+^ T cells in Yeti mice (Supplementary Figure 2). In muscle damage mediated by idiopathic inflammatory myopathies, myoblast-derived IL15 induced the differentiation of naive CD8^+^ T cells into highly activated and cytotoxic NKG2D^high^CD8^+^ T cells, which promoted myoblast damage through NKG2D-dependent lysis induced by MHC class I chain-related molecules (MICA and MICB) (37). Moreover, in celiac disease, NKG2D^high^CD8^+^ T cells induced by IL15 displayed cytotoxicity against intestinal epithelial cells expressing high MIC levels in a TCR-independent and NKG2D-mediated cytolysis pathway (38). Recently, it has been reported that in acute hepatitis A patients, NKG2D^high^CD8^+^ T cells induced by IL15 promote damage of liver tissue expressing NKG2D ligands such as MICA and MICB (39). Because stress induces increased expression of MICA and MICB on human intestinal epithelial cell lines (40), enhanced expression of murine NKG2D ligands on intestinal epithelial cells of DSS-treated Yeti/CD1d KO mice might trigger NK1.1^+^CD8^+^ T cell activation, which in turn might exacerbate DSS-induced colitis. Moreover, Crohn's disease patients exhibit markedly increased expression of IL15 and IFNγ in the inflamed colon compared with healthy people (41), indicating that NK1.1^+^CD8^+^ T cells involved in intestinal inflammation in Yeti mice might be physiologically relevant to the development of Crohn's disease. It will be of interest to further investigate what types of cells might represent the human counterpart for murine NK1.1^+^CD8^+^ T cells.

It has been reported that repression of IFNγ mRNA decay leads to accumulation of self-reactive CD8^+^ T cells, causing pancreas-specific damage (42). In addition, a recent study showed that IFNγ production by CD4^+^ T cells from homozygous Yeti mice is regulated by lactate dehydrogenase A (LDHA) via a 3′ UTR-independent mechanism, whereas IFNγ production by NK cells was not affected by these pathways (43). Consistent with the previous report that IFNγ mRNA transcripts are maintained constitutively in NK receptor-expressing innate immune cells such as NK cells and NKT cells (44), we demonstrated that NK1.1^+^CD8^+^ T cells expressing NK receptors constitutively produced large amounts of IFNγ. Thus, our results suggest that NK1.1^+^CD8^+^ T cells might be one of the main cellular sources of increased IFNγ in Yeti mice, in which the stability of IFNγ mRNA transcripts is enhanced.

A previous report revealed that IL15-induced NK1.1^+^CD8^+^ T cells display downregulated levels of TGFβ receptor, implying that NK1.1^+^CD8^+^ T cells are inherently resistant to immune suppression mediated by Treg-derived TGFβ (45). In addition, our data showed that NK1.1^+^CD8^+^ T cells could strongly attenuate Treg development via IFNγ signaling. These findings suggest that IFNγ-producing NK1.1^+^CD8^+^ T cells cannot respond to Treg cells and instead might inhibit Treg functions in the intestine.

In conclusion, the overall severity of DSS-induced colitis is determined by the extent of imbalance between protective and pathogenic cells and factors. Since our results clearly showed that iNKT cells are required for controlling NK1.1^+^CD8^+^ T cell-mediated pathogenesis during DSS-induced colitis, glycolipid antigens might be employed for designing more effective and safer therapeutics for IBD. Since iNKT cells are known to have subsets (iNKT1, iNKT2, and iNKT17), in future studies, it will be of interest to determine which subsets of iNKT cells contribute to the protective effect against NK1.1^+^CD8^+^ T cell-mediated intestinal inflammation. It will also be important to confirm such immunoregulatory roles of iNKT cells by employing more specific iNKT cell-deficient mouse model, such as the strain recently generated by CRISPR/Cas9 technology (46).

## Author contributions

SL: study concept and design, acquisition of data, analysis and interpretation of data, drafting of the manuscript, statistical analysis. HP: study concept and design, acquisition of data, analysis and interpretation of data, drafting of the manuscript, statistical analysis, obtained funding. JC: interpretation of data and review of the manuscript, obtained funding. LW: interpretation of data and review of the manuscript. LVK: interpretation of data and drafting of the manuscript, review of the manuscript. SH: study concept and design, acquisition of data, analysis and interpretation of data, drafting manuscript, statistical analysis, obtained funding, administrative, and material study supervision.

### Conflict of interest statement

The authors declare that the research was conducted in the absence of any commercial or financial relationships that could be construed as a potential conflict of interest.
