# Supplementary material for: iNKT Cells Suppress Pathogenic NK1.1+CD8+ T Cells in DSS-Induced Colitis
Source: Front Immunol. 2018 Oct 2;9:2168. doi: 10.3389/fimmu.2018.02168 (PMC6176072; doi:10.3389/fimmu.2018.02168)
Supplement: Supplementary file 1 [file Data_Sheet_1.docx]

**Supporting documents to:**

**iNKT cells suppress pathogenic NK1.1^+^CD8^+^ T cells**

**in DSS-induced colitis**

**This document includes:**

**-Supplementary figures 1-2**

**
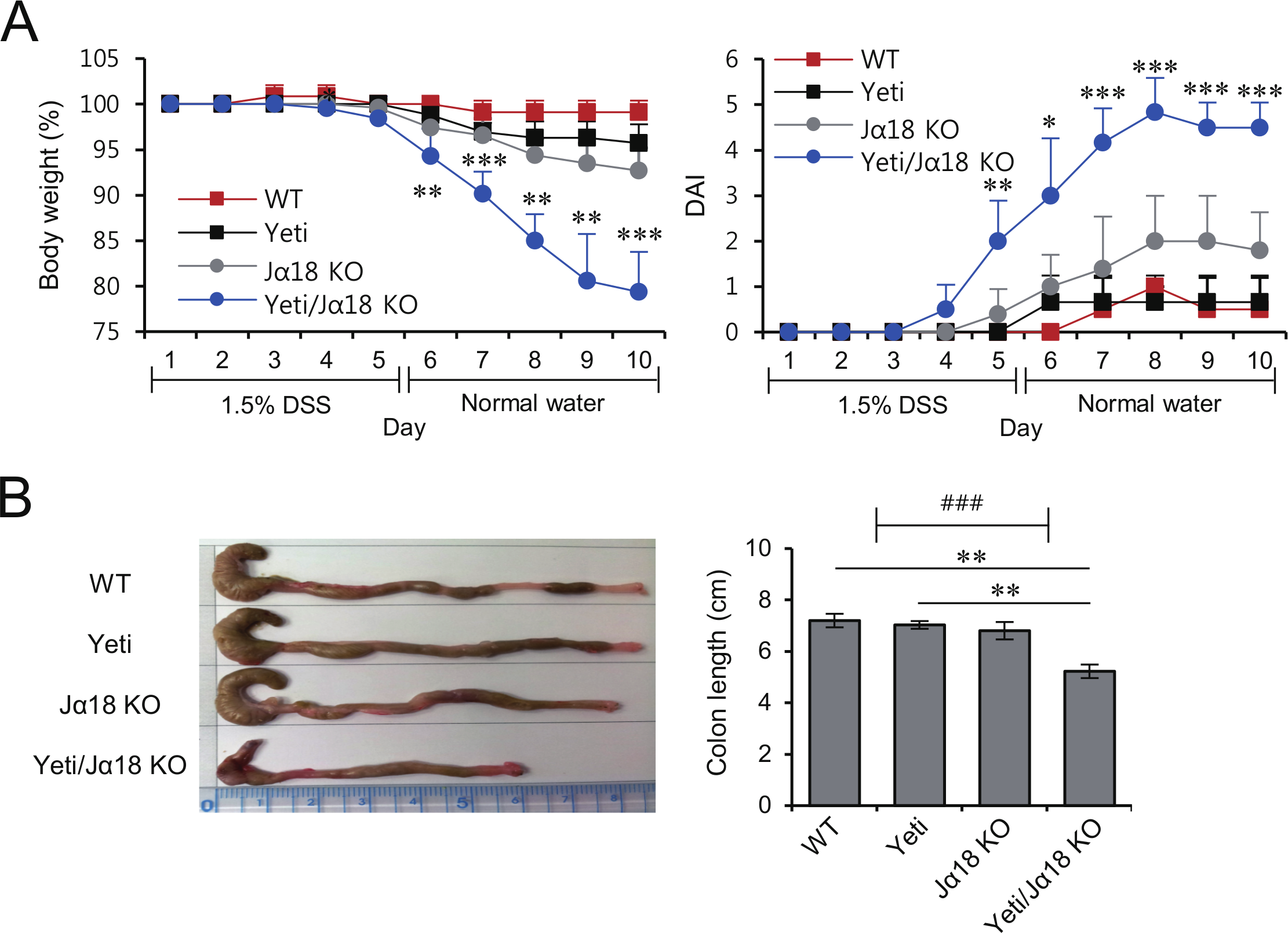
**

**Supplementary Figure 1.** Lack of iNKT cells accelerates intestinal inflammation in Yeti mice.

(A-B) Daily body weight changes, DAI score (A) and colon length (B) of WT, Yeti, Jα18 KO, and Yeti/ Jα18 KO mice were evalulated after 1.5% DSS treatment. Data are representative of two independent experiments with similar results. The mean values ± SD (n=5 per group in the experiment; Student’s t-test; *P<0.05, **P<0.01, ***P<0.001) are shown. Two-way ANOVA (Yeti × iNKT and genotype × tissue) showed an interaction between these two factors (^###^P<0.001).

**
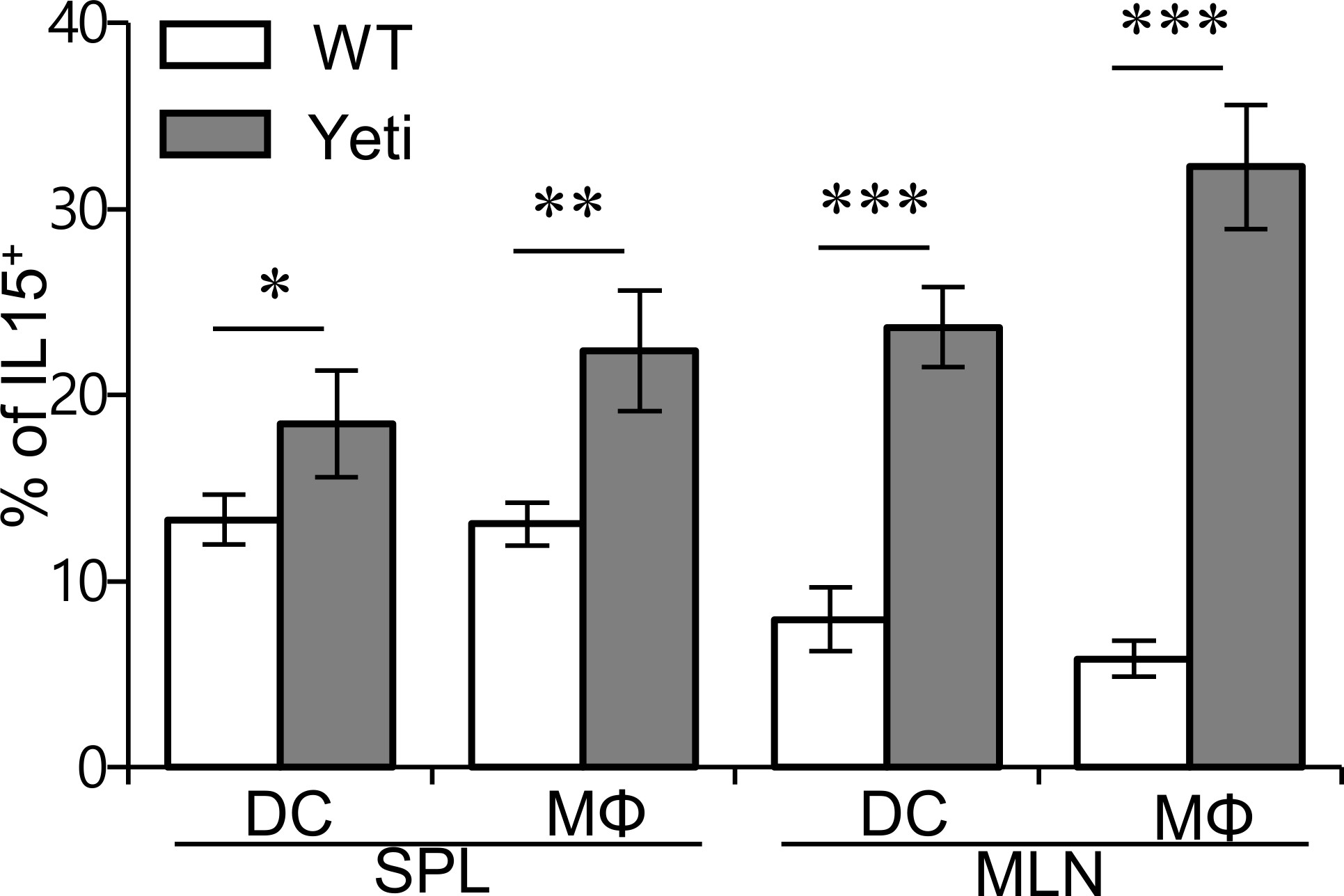
**

**Supplementary Figure 2.** Yeti mice display increased IL15 production by DCs and macrophages in the spleen and MLN.

Splenocytes and MLN cells from WT or Yeti mice were stimulated with PMA/Ionomycin for 1 hour and intracellular expression of IL15 by DCs and macrophages in the spleen or MLN was determined by flow cytometry. The mean values ± SD (*n* = 4 in C; per group in the experiment; Student’s t-test; *P<0.05, **P<0.01, ***P<0.001) are shown.
